# The effects of moderate alterations in adrenergic activity on acute appetite regulation in obese women: A randomised crossover trial

**DOI:** 10.1177/0260106020942117

**Published:** 2020-07-30

**Authors:** Fotini Tsofliou, Yannis P Pitsiladis, Jose Lara, Marios Hadjicharalambous, Ian A Macdonald, Mike A Wallace, Mike E J Lean

**Affiliations:** 1 Human Nutrition, School of Medicine, 3526 University of Glasgow, United Kingdom; 2 Department of Rehabilitation and Sport Sciences, Faculty of Health and Social Sciences, 375756 Bournemouth University, United Kingdom; 3 College of Medical Veterinary and Life Science, Institute of Cardiovascular & Medical Sciences, 3526 University of Glasgow, United Kingdom; 4 Centre for Sport and Exercise Science and Medicine, University of Brighton, United Kingdom; 5 Department of Applied Sciences, Faculty of Health and Life Sciences, 5995 Northumbria University, United Kingdom; 6 Human Performance Laboratory, Department of Life & Health Sciences, School of Sciences and Engineering, 121343 University of Nicosia, Cyprus; 7 School of Life Sciences, 6123 University of Nottingham Medical School, Queen’s Medical Centre, United Kingdom; 8 University Department of Pathological Biochemistry, Glasgow Royal Infirmary, United Kingdom

**Keywords:** Appetite regulation, adrenaline infusion, adrenergic blockade, moderate exercise, obesity

## Abstract

**Background:**

Previous evidence has demonstrated that serum leptin is correlated with appetite in combination with, but not without, modest exercise.

**Aim:**

The present experiments investigated the effects of exogenous adrenaline and α/β adrenoceptor blockade in combination with moderate exercise on serum leptin concentrations, appetite/satiety sensations and subsequent food intake in obese women.

**Methods:**

A total of 10 obese women ((mean ± SEM), age: 50 (1.9) years, body mass index 36 (4.1) kg/m^2^, waist 104.8 (4.1) cm) participated in two separate, double-blind randomised experimental trials. Experiment 1: moderate exercise after α/β adrenergic blocker (labetalol, 100 mg orally) versus moderate exercise plus placebo; experiment 2: adrenaline infusion for 20 minutes versus saline infusion. Appetite/satiety and biochemistry were measured at baseline, pre- and immediately post-intervention, then 1 hour post-intervention (i.e., before dinner). Food intake was assessed via ad libitum buffet-style dinner.

**Results:**

No differences were found in appetite/satiety, subsequent food intake or serum leptin in any of the studies (experiment 1 or experiment 2). In experiment 1, blood glucose was higher (*p* < 0.01) and plasma free fatty acids lower (*p* = 0.04) versus placebo. In experiment 2, plasma free fatty acids (*p* < 0.05) increased after adrenaline versus saline infusion.

**Conclusions:**

Neither inhibition of exercise-induced adrenergic activity by combined α/β adrenergic blockade nor moderate increases in adrenergic activity induced by intravenous adrenaline infusion affected acute appetite regulation.

## Introduction

Obesity is the most prevalent single disease in the world (International Classification of Diseases 10 code E.66), with more than 2.1 billion overweight adults (Ng et al., 2013). Better understanding of the mechanisms that regulate food intake, energy expenditure (EE) and energy balance is critical for the prevention and management of obesity. Physical activity has been implicated in appetite and body mass regulation; appetite also seems to be ‘coupled’ with body weight control in individuals undertaking moderate physical activity (Shook et al., 2015). Although physical activity tends to increase food intake (Westerterp et al. 2015), habitual exercisers are able to closely match food intake to EE (Martins et al., 2008); however, the mechanism underpinning the coupling between physical exercise and food intake regulation has yet to be explained.

In experimental rodent models and in cases of congenital obesity, leptin is a key regulator promoting satiety (Farooqi et al., 2009). In humans, leptin concentration is closely correlated to total fat mass (FM; Considine et al., 1996) and physical activity strongly predicts circulating leptin concentrations independently of body FM, suggesting a plausible role of physical activity in leptin sensitivity (Chu et al., 2001). Raised circulating leptin concentrations do not appear to prevent overeating in obese humans, who are considered ‘leptin resistant’ (Lean and Malkova, 2016). Indeed, most models of diet-induced obesity in rodents have presented evidence that obesity causes central and peripheral leptin resistance whereby anorexigenic/orexigenic neurons fail to signal satiety in response to high circulating leptin (Morris and Rui, 2009). Leptin’s transport across the blood-brain barrier is also reduced concurrently with increasing adiposity (Banks and Farrell, 2003). As human obesity is associated with impaired appetite control, this implies that other factors may influence the anorexic effects of leptin.

Several studies have demonstrated the acute regulation of circulating leptin turnover by adrenergic agents and catecholamine (Keller et al., 2005; Scriba et al., 2000) and the role of endogenous catecholamine in the hypothalamic paraventricular nucleus (PVN) has been related to eating or satiety (Wellman, 2000). For example, activation of α2-adrenoceptors in the PVN enhances eating, whereas activation of α1-adrenoceptors inhibits eating (Wellman et al., 1993). Moreover, an acute effect of elevated adrenaline levels on enhanced leptin transport into the brain through activation of predominantly α1-adrenoceptors was found in rats (Banks, 2001). A link between obesity, inactivity and raised circulating leptin concentrations has been clearly demonstrated (Chu et al., 2001), which suggests that high circulating leptin concentrations are ineffective in regulating appetite and body mass when physically inactive. Studies in lean and obese rats suggested that acute and chronic exercise improved the antiorexigenic action of leptin, as well as hypothalamic leptin signalling (Krawczewski et al., 2011; Ropelle et al., 2010).

We also reported an association between circulating leptin and appetite suppression in obese individuals, but only following an acute bout of moderate-intensity exercise (Tsofliou et al., 2003). These studies support a role of exercise in mediating the action of leptin on appetite regulation in the short term. As even light exercise is known to produce a marked stress response in sedentary individuals (Salvadori et al., 2003), the increase in catecholamines that normally accompanies such a response might be responsible for the coupling of leptin and appetite. Adrenaline may facilitate leptin transport into the brain through stimulation of α-adrenoceptors located at the blood side of the blood-brain barrier (Banks, 2001). The purpose of the current study was to investigate the effects of increased circulating adrenaline concentrations by exogenous intravenous administration and the effects of moderate exercise performed during α/β-adrenoceptor blockade on our primary outcomes, appetite-satiety measures and on subsequent food intake in obese women. We also investigated the impact of these interventions on biological markers such as circulating leptin, glucose and free fatty acid (FFA) concentrations, using the association between serum leptin and appetite/satiety sensations as an indirect index of leptin sensitivity.

### Material and methods

This study is reported according to the CONSORT guidelines (Schulz et al., 2010) (Figure S1 and Table S1 in Supplementary Files).

### Participants

A total of 10 (*n* = 10) obese but otherwise healthy, premenopausal women (Table 1) gave written informed consent to participate in the study, which was conducted in accordance with the Declaration of Helsinki. The sample size used in this study was based on the primary outcomes of interest such as appetite ratings and ad libitum intake. Using a paired design and a power of 0.8, a minimum of nine participants would be needed to detect a 10 mm difference in postprandial ratings and to detect a 100 kcal difference in ad libitum energy intake (EI) (Lara et al., 2010; Horner et al., 2014). The protocol was approved by the Glasgow Royal Infirmary Research Ethics Committee (01HU009, 02HU002). All participants were in good physical and mental health with normal blood pressure (≤ 140 / ≤ 90 mmHg), non-smokers, on no medication known to affect appetite, not known to be anaemic or hyperlipidemic and not on a special diet. Following eligibility screening and familiarisation with methodological procedures, using a double-blind, crossover design, participants were randomised to the intervention for each experiment (EXP-1 and EXP-2) using an online random number generator (http://www.randomization.com). The order of the trials for each experiment was randomised separately. There was an interval of at least 7 days between trials. In EXP-1 (exercise with either α/β-adrenoceptor blocker or placebo) all 10 eligible participants took part in the study procedures and data analysis whereas in EXP-2 (adrenaline vs saline infusion) results are presented from nine participants; one participant did not continue after EXP-1.

**Table 1.** Subject characteristics, *n* = 10.

Age (years)	50.3 ± 1.9
Weight (kg)	90.2 ± 5.2
Height (cm)	158.0 ± 0.02
BMI (kg^.^m^−2^)	36.0 ± 4.1
Waist circumference (cm)	104.8 ± 4.1
Hip circumference (cm)	115.2 ± 3.1
Fat mass (%) predicted by waist measurement	47.7 ± 1.7
Systolic blood pressure (mmHg)	129.6 ± 2.4
Diastolic blood pressure (mmHg)	89.2 ± 1.4

Values are mean ± SEM.

BMI: body mass index.

Concealed treatment allocation was implemented; a person, unrelated to the trial, prepared the treatment allocation using sealed opaque envelopes. Both participants and researchers evaluating the impact of the experiments were blinded to treatment. Intervention agents were dispensed at each visit by two members of the staff not involved in the study.

## Experimental design and procedures

**Figure 1.**
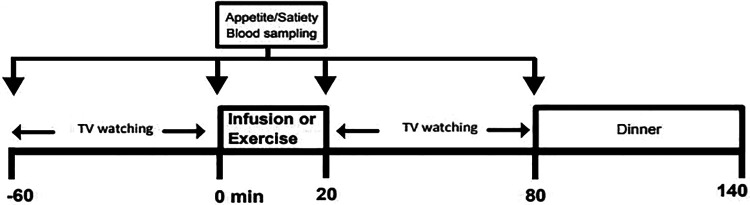
Schematic representation of the study design, EXP 1: Moderate exercise plus adrenergic blocker vs. placebo for 20 min; EXP 2: Adrenaline vs. Saline infusion for 20 min.

Adrenaline was infused (MacCarthy and Bloomfield, 1983; Centers for Disease Control, 2014), raising circulating adrenaline levels to those typically seen during moderate exercise (Lean et al., 1996). On a separate occasion, labetalol, which blocks α_1_-, β_1_- and β_2_-adrenoceptors (MacCarthy and Bloomfield, 1983; McLoughlin et al., 1992) was administered prior to moderate exercise. Participants visited the laboratory on four occasions to participate in four acute interventions with an interval of at least 7 days between trials (Figure 1); EXP-1 involved moderate-intensity exercise with either α/β-adrenoceptor blocker or placebo and EXP-2 adrenaline infusion versus saline infusion. Participants kept diet and physical activity records for 2 days preceding the first experimental trial. These food and activity patterns were replicated before all subsequent trials. Household measures (e.g., glasses, cupfuls, tablespoons, slices, etc.) were used to quantify food and fluid consumption. For each experiment, participants visited the laboratory approximately 5 hours after a standard lunch and the time duration was standardised within subject. On arrival at the laboratory, weight, waist and hip circumference were measured using calibrated scales and inextensible tapes with bone landmarks for anthropometry (Centers for Disease Control, 2014). The body fat percentage was predicted from waist (Lean et al., 1996). Arterialised-venous blood samples (McLoughlin et al., 1992) were collected from an 18G indwelling catheter placed by percutaneous puncture into a vein on the dorsum of a heated hand and a baseline sample (-60 minutes) was taken. Serial blood samples (10 ml) were then drawn at 0, 20 and 80 minutes. Following each blood sample, participants completed a set of self-rating 100 mm visual analogue scales for hunger, desire to eat, prospective food consumption (PFC), satiety and fullness (Stubbs et al., 2000). Throughout each trial, participants were seated in a comfortable environment watching food-related digital versatile DVDs for 60 minutes. Food-related DVDs were intended to direct participants’ attention towards food and eating, to stimulate a familiar form of home entertainment that might reduce anxiety and eating restraint (Bellisle and Dalix, 2001).

After watching the food-related DVDs, participants took part in one of the following interventions on each of the 4 study days. In EXP-1, 60 minutes prior to each of the two exercise trials, participants were given either 100 mg labetalol (Generics UK) or placebo (calcium carbonate). Then the participants were required to walk at a moderate pace (5 km/h) on a motorised treadmill for 20 minutes. This is in line with a previous study of our group that found acute leptin coupling with appetite/satiety measures after a bout of moderate intensity exercise in obese women (Tsofliou et al., 2003).

In EXP-2, a single dose of either adrenaline hydrochloride (i.e., 1:10,000) diluted in normal saline, or normal saline alone was infused intravenously at a rate of 12.5 ng min/kg ideal body mass, via a pump for 20 minutes (Webber et al., 1994) to yield a plasma level not exceeding 1 nmol/L. This dosage ensures the plasma catecholamine concentration will not exceed the level typically measured following moderate-intensity exercise (Gustafson et al., 1990). This dosage aimed to maintain catecholamine concentrations similar to the levels attained by the 20 minutes of moderate exercise (McLoughlin et al., 1992). The DVD was switched off for 20 minutes during each infusion.

Following each intervention, participants continued watching food-related DVDs for another hour. They were then offered a buffet-type dinner comprising 11 food items: roasted chicken breast (200 g), roasted baby potatoes (160 g), onion stuffing (60 g), boiled peas (126 g), boiled carrots (116 g), boiled corn (118 g), tuna cucumber sandwich (176 g), chicken and salad sandwich (178 g), banana (100 g), two apple pies (120 g), potato crisps (26 g) and orange juice (500 ml) and were told to eat as much as they wanted within 1 hour. Each person’s selection from the buffet dinner was analysed for EI and macronutrient content using a computerised version of McCance and Widdowson’s (revised by Holland et al., 1993) food composition tables and relative EI (REI) was calculated for both exercise trials in EXP-1 as EI minus the energy cost of the exercise (Douglas et al., 2015).

Rating of perceived exertion (breathlessness and leg exertion) (Borg, 1982) and heart rate (HR) (Polar Sport Tester, Polar Electro Oy, Finland) were recorded every 10 minutes during the moderate exercise and the infusion interventions. For EXP-1, expired gas was collected in Douglas bags for 5 minutes at rest, and thereafter 1 minute collections were obtained every 10 minutes during the moderate exercise interventions. Expired gases were analysed within 5 minutes of collection for O_2_ (Servomex 570A, East Sussex, UK) and CO_2_ (Servomex 1400 B4, East Sussex, UK), volume (dry gas meter, Harvard Apparatus Ltd., Hertfordshire, UK) and temperature (C6600 10-Channel Microprocessor, Comark, Hertfordshire, UK). Barometric pressure was measured using a standard mercury barometer. Oxygen uptake (VO_2_), carbon dioxide production (VCO_2_) and respiratory exchange ratio (RER, i.e., VO_2_/VCO_2_) were subsequently evaluated and the percentages of fuel oxidation were determined. EE (kcal·min^−1^) (Ravussin et al., 1985) and the rates of fat and carbohydrate oxidation (g·min^−1^) (Alkahtani, 2014) were calculated by standard equations: EE = {4.686 + [(RER − 0.707) / 0.293] x 0.361} x VO_2_; fat oxidation = (1.67 x VO_2_) − (1.67 x VCO_2_); carbohydrate oxidation = (4.55 x VCO_2_) − (3.21 x VO_2_).

### Blood treatment and analyses

Venous blood was collected in K3EDTA vacutainers for the measurement of blood glucose, plasma free fatty acids (FFA) (colorimetric method, Boehringer Mannheim Biochemica, London, UK) and into clot activator vacutainers for serum leptin measurement. Duplicate aliquots (400 µl) of whole blood from the K3EDTA tube were rapidly deproteinised in 800 µl of 0.3 mol^.^l^−1^ perchloric acid; following centrifugation the supernatant was used for the measurement of glucose (Maughan, 1982). Plasma supernatant was separated and plasma (500 μl) was mixed with 50 µl EGTA-glutathione and stored at -70°C for subsequent determination of adrenaline and noradrenaline (NA) (Forster and Macdonald, 1999). The remaining plasma was stored at −20°C and later used for the measurement of FFA (colorimetric method, Boehringer Mannheim Biochemica, London, UK). Blood collected in the clot activator vacutainer was allowed to clot for 10 minutes. Following centrifugation, the serum was stored at -70°C and subsequently analysed for leptin by radioimmunoassay.

### Statistical analysis

Statistical analyses were carried out with IBM SPSS v22 for Windows. To assess the impact of interventions, statistical analysis of the data was carried out using General Linear Model with repeated measures followed by pairwise analysis with Bonferroni adjustment. Results are presented as estimated marginal means ± SEM. Correlation analysis was also carried out between the serum leptin concentrations and appetite measures (for each time point separately) and adiposity indices. Statistical significance was taken as *p* < 0.05.

## Results

### Effects on self-reported appetite-satiety ratings and subsequent dietary intake

**Figure 2.**
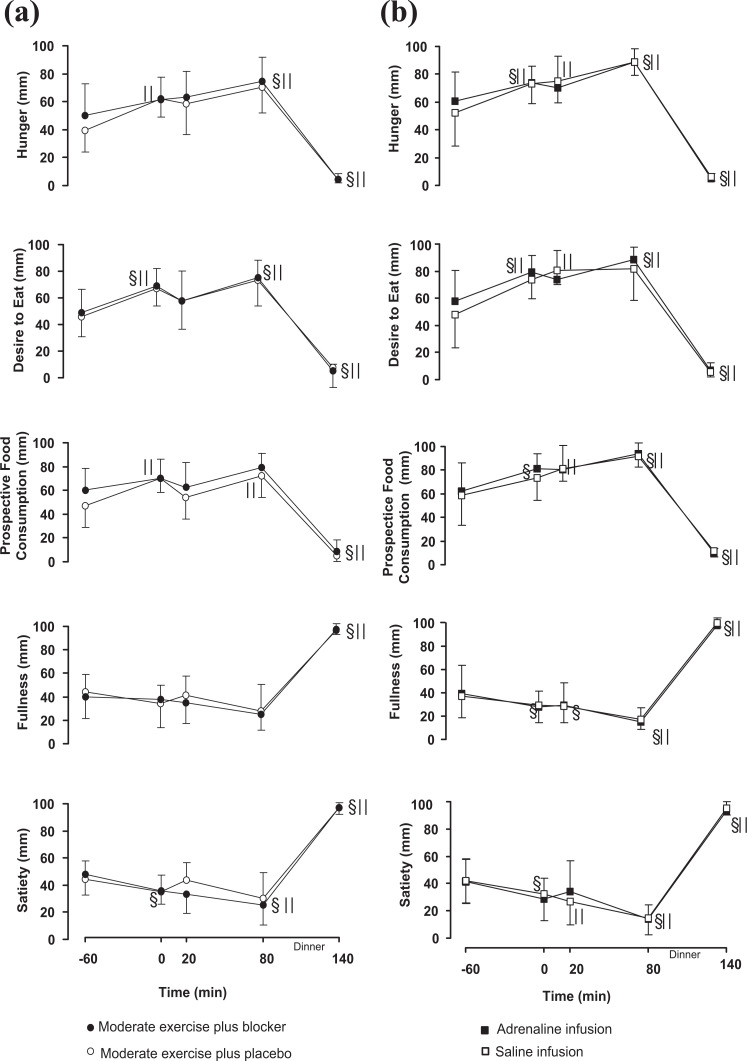
Mean ± SEM profiles of self-reported appetite-satiety ratings in the Moderate exercise plus a/b blocker (•) and Moderate exercise plus placebo (ˆ) trials (2a, left side), and in the Adrenaline infusion (▪) and Saline infusion (□) trials (2b, right side); ^§||^: are significantly different from baseline within the Moderate exercise plus a/b blocker (•^§^) or the Moderate exercise plus placebo (ˆ^||^) trial and within the Adrenaline infusion (▪^§^) or the Saline infusion trials (□^||^), *p* < 0.001.

Profiles of hunger, desire to eat, PFC, fullness and satiety throughout each intervention in both experiments are shown in Figures 2(a) and 2(b). In both EXP-1 and EXP-2, a main time effect was observed in all appetite-satiety measures and there were no significant differences on appetite/satiety measures between interventions.

In EXP-1, the general linear model showed a significant time effect for hunger ratings (*p* = 0.003), satiety, desire to eat and for PFC ratings (*p* = 0.002). No differences were found over time in PFC or fullness ratings (Figure 2(a), left side). In EXP-2 there was a significant time effect for hunger, satiety, fullness, PFC and for the desire to eat (*p* < 0.001) (Figure 2(b), right side).

Self-selected food intake at dinner did not differ significantly between trials in either EXP-1 or EXP-2 (Table 2).

**Table 2.** Buffet-style dinner intake subsequent to all interventions.

Dietary intake	Exercise plus placebo, *n* = 10	Exercise plus α/β blocker, *n* = 10	*p v*alue	Adrenaline infusion, *n* = 9	Saline infusion, *n* = 9	*p v*alue
Energy intake (kcal)	812.7 ± 75.9	899.9 ± 64.7	0.23	1023.3 ± 81.2	1013.2 ± 79.7	0.85
Protein (g)	57.1 ± 6.6	59.2 ± 5.5	0.48	67.8 ± 7.6	65.2 ± 6.1	0.43
Protein (%)	28 ± 1.6	27 ± 2.3	0.67	26.5 ± 2.2	27 ± 2.1	0.92
Carbohydrate (g)	103.4 ± 8.2	112.8 ± 7.9	0.41	124.9 ± 9.6	120.2 ± 11.5	0.43
Carbohydrate (%)	50 ± 2.7	48 ± 3.1	0.62	47 ± 2.8	45 ± 1.7	0.10
Fat (g)	21.5 ± 2.7	26.1 ± 3.6	0.17	31.3 ± 3.4	33.1 ± 3.4	0.48
Fat (%)	22 ± 1.5	25 ± 6.8	0.30	26± 1.3	28 ± 4.2	0.06

Data are shown as mean ± SEM; no significant differences between interventions in both EXP-1 and EXP-2 (paired *t*-test).

EXP: experiment.

### Effects on biochemical measures in both experiments

In EXP-1 there was no effect of intervention (*p* = 0.6) and time by intervention interaction (*p* = 0.4) for serum leptin. Significant differences were found in blood glucose and plasma FFA between the two moderate exercise interventions. Blood glucose concentrations were significantly higher and plasma FFA was significantly lower for 1 hour after the moderate exercise plus α/β blocker intervention compared to exercise plus placebo (Table 3).

**Table 3.** Serum leptin, blood glucose, plasma FFA during EXP-1, *n* = 10.

	Interventions	(-60 min)	(0 min)	(20 min)	(80 min)	Time	Intervention	Intervention x time
Serum leptin (ng^.^ml^−1^)	Exercise plus placebo	62.28± 6.99	65.71 ± 8.39	73.01 ± 8.45	65.65 ± 7.41	*p* = 0.0004	*p* = 0.694	*p* = 0.406
	Exercise plus α/β blocker	62.75± 7.27	63.37 ± 7.33	68.90 ± 7.5	65.24 ± 7.84			
Blood glucose (mmol^.^l^−1^)	Exercise plus placebo	4.63 ± 0.16	4.53 ± 0.08	4.55 ± 0.09	4.52 ± 0.06	*p* = 0.659	*p* = 0.0004	*p* = 0.028
	Exercise plus α/β blocker	4.59 ± 0.16	4.83 ± 0.11	4.91 ± 0.07^a^	4.89 ± 0.06^a^			
Plasma FFA (mmol^.^l^−1^)	Exercise plus placebo	0.61 ± 0.13	0.65 ± 0.08	0.74 ± 0.09	0.73 ± 0.07	*p* = 0.866	*p* = 0.101	*p* < 0.001
	Exercise plus α/β blocker	0.67 ± 0.11	0.59 ± 0.07	0.49 ± 0.06^a^	0.59 ± 0.06^a^			

Values are estimated marginal means ± SEM. Analysis was conducted by GLM with repeated measures adjusted for multiple comparisons using Bonferroni corrections.

^a^ Significant differences between exercise interventions (exercise plus α/β blocker vs exercise plus placebo: glucose 20 minutes, *p* = 0.001, 80 minutes (after dinner) *p* < 0.001; FFA 20 minutes, *p* = 0.02, 80 minutes (after dinner) *p* = 0.005).

EXP: experiment; FFA: free fatty acids; GLM: General Linear Model.

In EXP-2 there was no significant difference on serum leptin concentrations and blood glucose concentrations between the adrenaline and the saline infusions or over time, throughout the trials (*p* > 0.05). Plasma concentrations of FFA were significantly higher immediately after the adrenaline infusion compared to saline infusion (FFA, *p* = 0.032). In addition, plasma NA concentrations showed a borderline significant difference between treatments (Table 4).

**Table 4.** Serum leptin, blood glucose, plasma FFA, plasma adrenaline and noradrenaline (NA) concentrations during the EXP-2, *n* = 9.

						*p v*alue		
	Interventions	(-60 min)	(0 min)	(20 min)	(80 min)	Time	Intervention	Intervention x Time
Serum leptin (ng^.^ml^−1^)	Adrenaline infusion	63.68 ± 7.77	63.20 ± 8.11	61.98 ± 8.58	67.70 ± 10.49	*p* = 0.068	*p* = 0.688	*p* = 0.961
	Saline infusion	65.80 ± 8.15	65.86 ± 8.07	65.31 ± 9.18	68.90 ± 7.73			
Blood glucose (mmol^.^l^−1^)	Adrenaline infusion	4.79 ± 0.34	4.59 ± 0.09	4.76 ± 0.82	4.530 ± 0.07	*p* = 0.136	*p* = 0.696	*p* = 0.532
	Saline infusion	5.03 ± 0.27	4.72 ± 0.06	4.60 ± 0.05	4.575 ± 0.03			
Plasma FFA (mmol^.^l^−1^)	Adrenaline infusion	0.75 ± 0.15	0.84 ± 0.13	1.09 ± 0.17^a^	0.82 ± 0.11	*p* = 0.010	*p* = 0.025	*p* = 0.083
	Saline infusion	0.56 ± 0.11	0.57 ± 0.13	0.65 ± 0.15	0.70 ± 0.10			
Plasma adrenaline (nmol^.^l^−1^)	Adrenaline infusion	–	0.17 ± 0.26	–	–			
	Saline infusion	–	0.16 ± 0.20	–	–			
Plasma NA (nmol^.^l^−1^)	Adrenaline infusion	–	1.59 ± 0.19	2.32 ± 0.19	–	*p* = 0.010	*p* = 0.063	*p* = 0.060
	Saline infusion	–	1.49 ± 0.26	1.61 ± 0.26	–			

Values are estimated marginal means ± SEM. Analysis was conducted by ANOVA with repeated measures adjusted for multiple comparisons using Bonferroni corrections.

^a^ Significant differences between infusion trials (adrenaline infusion vs saline infusion: at 20 min FFA; *p* = 0.032) (pairwise comparisons, adjustment for multiple comparisons: Bonferroni). Post-adrenaline infusion values of circulating adrenaline concentrations were not determined due to unresolved co-eluting peaks with adrenaline.

ANOVA: analysis of variance; FFA: free fatty acids.

Baseline serum leptin concentrations correlated significantly with body mass index (BMI, kg^.^m^−2^), FM (%) and waist circumference (BMI *r* = 0.78, *p* = 0.01, FM *r* = 0.63, *p* = 0.04, waist *r* = 0.71, *p* = 0.02). No significant associations were found between serum leptin concentrations and appetite-satiety measures at any time point in the two experiments (*p* > 0.05).

### Physiological responses to treadmill walking and adrenaline infusion

HR, perceived breathlessness and leg tiredness during the moderate exercise and the infusion interventions are show in Table 5; there was no significant difference in HR between trials in either EXP-1 or EXP-2 (Table 5). The average EE of participants was 136 kcal (± 30) and 128 (± 40) in exercise plus placebo and exercise plus α/β blocker respectively; the EE was not significantly different between exercise trials. In both EXP-1 and EXP-2, oxygen uptake (VO_2_), carbon dioxide production (VCO_2_), RER and fuel Fat, Carbohydrate (CHO) oxidation rates were not significantly different between trials (Table 6).

**Table 5.** Heart rate, perceived breathlessness and leg-tiredness during the exercise and infusion interventions in both experiments.

			Time (min)				*p*-value		
	Interventions	Rest	5	10	15	20	Time	Intervention	Intervention x time
Heart rate (beats.min^−1^)	Exercise plus placebo	79.83 ± 7.39	121.33 ± 9.93	132.17 ± 11.13	131.17 ± 8.87	134.33 ± 10.94	<0.001	0.572	0.146
	Exercise plus α/β blocker	86.50 ± 6.24	119.83 ± 18.43	128.17 ± 10.62	125.83 ± 7.56	130.67 ± 11.02			
	Adrenaline infusion	76.60 ± 5.28	75.40 ± 4.93	78.00 ± 4.95	81.20 ± 3.63	81.80 ± 3.99	0.016	0.098	0.053
	Saline infusion	76.00 ± 5.21	73.50 ± 4.87	75.00 ± 6.04	74.20 ± 5.61	75.40 ± 4.53			
Perceived breathlessness (rating 0–20)	Exercise plus placebo	7.83 ± 0.70	9.83 ± 0.54	11.17 ± 0.65	11.50 ± 0.81	12.33 ± 0.53	<0.001	0.468	0.758
	Exercise plus α/β blocker	7.17 ± 0.17	10.00 ± 0.76	11.00 ± 0.67	12.17 ± 0.48	12.33 ± 0.33			
	Adrenaline infusion	8.29 ± 0.78	7.71 ± 0.64	8.00 ± 0.66	7.71 ± 0.64	7.71 ± 0.644	0.461	0.458	0.394
	Saline infusion	7.86 ± 0.63	8.00 ± 0.66	8.00 ± 0.66	7.86 ± 0.63	7.857 ± 0.634			
Perceived leg-tiredness (rating 0–20)	Exercise plus placebo	7.33 ± 0.42	10.67 ± 0.67	11.50 ± 0.56	12.33 ± 0.72	12.50 ± 0.34	<0.001	0.475	0.490
	Exercise plus α/β blocker	8.00 ± 0.63	10.33 ± 0.61	11.83 ± 0.83	12.83 ± 0.60	13.33 ± 0.76			
	Adrenaline infusion	7.50 ± 0.46	7.50 ± 0.46	7.75 ± 0.62	7.63 ± 0.53	7.63 ± 0.53	0.252	0.039	0.732
	Saline infusion	8.50 ±0.66^a^	8.75 ±0.73^a^	8.75 ± 0.73	8.75 ± 0.73^a^	8.75 ± 0.73^a^			

Values are estimated marginal means ± SEM. Analysis was conducted by General Linear Model (GLM) with repeated measures adjusted for multiple comparisons using the Bonferroni corrections.

^a^ Significant differences between infusion interventions (adrenaline infusion versus saline infusion: perceived leg-tiredness (rest *p* = 0.033, 5 min, 15 min and 20 min, *p* = 0.038).

**Table 6.** Gas exchange, energy expenditure and substrate oxidation in EXP-1 (at rest and during 20 min of exercise) and in EXP-2 (at rest and during 20 min of adrenaline/saline infusion.)

	Trials	Rest	20 min intervention
VO_2_ (L.min^−1^)	Exercise plus placebo	0.3 ± 0.04	1.4 ± 0.3
	Exercise plus α/β blocker	0.3 ± 0.06	1.3 ± 0.4
	Saline infusion	0.2 ± 0.09	0.3 ± 0.05
	Adrenaline infusion	0.3 ± 0.06	0.3 ± 0.05
VCO_2_ (L.min^−1^)	Exercise plus placebo	0.2 ± 0.06	1.1 ± 0.2
	Exercise plus α/β blocker	0.2 ± 0.07	1.1 ± 0.3
	Saline infusion	0.2 ± 0.07	0.2 ± 0.04
	Adrenaline infusion	0.2 ± 0.05	0.2 ± 0.03
Energy expenditure (kcal·min^−1^)	Exercise plus placebo	1.3 ± 0.1	6.8 ± 1.5
	Exercise plus α/β blocker	1.3 ± 0.3	6.4 ± 2.0
	Saline infusion	1.1 ± 0.4	1.2 ± 0.2
	Adrenaline infusion	1.3 ± 0.3	1.4 ± 0.2
CHO oxidation (g·min^−1^)	Exercise plus placebo	0.08 ± 0.33	0.58 ± 0.45
	Exercise plus α/β blocker	0.10 ± 0.15	0.55 ± 0.27
	Saline infusion	0.06 ± 0.15	0.07 ± 0.09
	Adrenaline infusion	0.08 ± 0.12	0.03 ± 0.15
Fat oxidation (g·min^−1^)	Exercise plus placebo	0.10 ± 0.13	0.48 ± 0.21
	Exercise plus α/β blocker	0.09 ± 0.04	0.45 ± 0.15
	Saline infusion	0.10 ± 0.06	0.15 ± 0.07
	Adrenaline infusion	0.10 ± 0.06	0.14 ± 0.07

Values are estimated marginal means ± SEM. No significant differences were found between trials in EXP-1 or EXP-2.

CHO: Carbohydrate; EXP: experiment.

## Discussion

In the current study, we examined the effects of exogenous adrenaline and α-/β-adrenoceptor blockade in combination with moderate exercise on serum leptin concentration, appetite/satiety sensations and food intake in obese women. It was envisaged that this approach would allow us to identify whether adrenergic stimulation mediates the central effect of leptin on appetite regulation. The novel result of the current study is that moderate manipulation of adrenergic activity via adrenaline infusion or α/β-adrenoceptor blockade using 100 mg labetalol during moderate-intensity exercise was not found to affect post-exercise appetite/satiety sensations and subsequent EI in obese women.

Previous studies have shown impaired catecholamine responses to physical exercise in obese individuals (Salvadori et al., 2003). In the current study, plasma NA concentration increased to 2.3 nmol·l^−^^1^ at the end of the adrenaline infusion (only borderline significance was found), typical of the suppressed levels found during exercise in obesity; substantial variation was reported in NA concentration during intense or exhaustive exercise in obese, young individuals (from 4.28 to 5.9 nmol·l^−1^) (Zouhal et al., 2013). HR tended to increase towards the end of the adrenaline infusion (82 b.min^−1^) at similar levels with previous adrenaline infusion studies in obese women (Walsh et al., 1998) but we did not observe significant differences; plasma FFA reached concentrations of 1.09 mmol·l^−1^, which is indicative of adrenaline-stimulated lipolysis (Webber et al., 1994). We were not able to determine post-adrenaline infusion values of circulating adrenaline concentrations due to unresolved peaks co-eluting with adrenaline. However, the plasma FFA profiles would be consistent with responses to plasma adrenaline concentrations above 0.6 nmol·l^−1^ (∼0.8 nmol·l^−1^ during 20 minutes of 12.5 ng per kg IBW (Ideal Body Weight) per minute adrenaline infusion), a level that would stimulate lipolysis (Webber et al., 1994).

Catecholamines have long been implicated in appetite regulation as clinical appetite suppressants in obese patients (Lean and Finer, 2006) and it is demonstrated that they exert regulatory effects on the expression of mRNA leptin and circulating leptin concentrations (Ricci and Fried, 1999). The current study is the first to investigate the role of short-term increases in adrenergic activity on the acute appetite response following exercise in humans. It was observed that 20 minutes of adrenaline infusion did not affect acute appetite or serum leptin concentration and leptin concentrations did not change after 20 minutes of moderate-intensity exercise. This is in agreement with others that found decreases in leptin only after prolonged moderate intensity exercise in trained men (Zaccaria et al., 2013) and overweight women (Tiryaki-Sonmez et al., 2013) or a delayed leptin reduction in active individuals within a 24 hour timeframe post-exercise (King et al., 2015). Notably, exercise-induced NA increase but not other biochemical factors (i.e., cortisol or FFA) was suggested to account for the reduction in post-exercise circulating leptin (Zaccaria et al., 2013). However, these studies either did not measure subsequent effects on appetite/satiety feelings post exercise or found no compensatory appetite response (King et al., 2015). As the exercise-induced appetite regulatory response, both hormonal and behavioural, might diverge in the presence of obesity (Heden et al., 2013), whether there is an interplay between adrenergic activity, leptin response and appetite expression after exercise remains to be clarified utilising different modes of exercise in individuals with different body weights.

Furthermore, research in physical exercise and appetite regulation has shown that single bouts of exercise might suppress the orexigenic ghrelin while simultaneously elevating anorexigenic signals peptide YY (PYY), glucagon-like peptide-1 (GLP-1), cholecystokinin (CCK) and pancreatic polypeptide (PP) (Zouhal et al., 2019). These observations have been reported mainly in lean, physically active males whereas evidence in females and particularly in individuals with obesity is sparse and contradictory. It is also suggested that exercise training in women with obesity might influence the regulation of food intake via improved leptin sensitivity (Martins et al., 2013). New evidence from animal studies indicates that leptin might enhance the effects of gut-satiety hormones, highlighting the importance of interactions among the feeding-related hormones that probably lead to an integrated anorectic signal (Akieda-Asai et al., 2014). Future studies need to measure leptin in conjunction with the other appetite-regulating peptides (acylated ghrelin, PYY, GLP-1, CCK and PP) to enable a better understanding of how exercise-induced responses to appetite-regulating hormones might differ in obesity (Dorling et al., 2018).

With regard to the effect of adrenaline infusion on acute appetite control in obese women, previous studies reported reduced circulating leptin concentrations after 60 minutes of adrenaline infusion (0.010 μg/kg fat free mass/min), suggesting that a decrease in obesity-related leptinemia could stimulate a compensatory appetite response but this was not assessed (Couillard et al., 2002). The lack of any significant adrenaline-induced decrease in serum leptin concentrations in the present study may be due to the shorter period of adrenaline infusion compared to previous studies, which found reduced circulating leptin levels after infusions of 60 to 180 minutes (Couillard et al., 2002). Secondly, the large variability in leptin response to adrenaline previously observed in human obesity, namely, low- and high-leptin responders, could account for the present unchanged leptin concentrations during adrenaline infusion and could indicate a potential heterogeneity in leptin sensitivity among obese individuals (Couillard et al., 2002). It is possible that adrenaline-induced changes in leptin could induce changes in appetite/satiety sensations and food intake in the short term, but additional work is necessary to understand the complexity of this physiological mechanism, the timeframe of its action and whether there are differences in the regulation of appetite and food intake between low- and high-leptin responders to adrenaline.

The current study was not able to reproduce the association between leptin and appetite sensations that was found in our earlier study (Tsofliou et al., 2003). There was no evidence for a difference in EI 1 hour after the moderate exercise with placebo (average 813 kcal) compared to α/β-adrenergic blockade (average kcal 900) (*p* = 0.2). When the REI was additionally calculated for the exercise trials, no difference in REI incurred between exercise with placebo (677 kcal) and exercise with α/β-adrenergic blockade (772 kcal). Previous data from walking studies reported no compensatory response in absolute EI in lean and obese individuals and no changes in relative EI or a significant decrease when the median energy deficit of exercise was around 335 kcal (Schubert et al., 2013). The present findings indicate that α/β-adrenergic blockade was not able to induce a different appetite response to exercise with placebo and did not trigger a compensatory response in EI and appetite sensations after an acute exercise-induced energy deficit. These findings, however, were derived from a small sample and require further verification.

In the present study, labetalol 100 mg resulted in a lower plasma FFA concentration immediately after and 1 hour after moderate exercise (0.49 nmol.l^−1^, 0.59 nmol.l^−1^ respectively) compared to placebo (0.74 nmol.l^−1^, 0.73 nmol.l^−1^ respectively) possibly by blocking the β-receptor mediated lipolysis (Ladage et al., 2013). The α/β-adrenergic blockade also induced a significant increase in post-exercise blood glucose concentration (4.9 mmol.l^−1^) compared to placebo (4.5 mmol.l^−1^). These results are supported by earlier studies (Hartling et al., 1980). However, they are disputed by recent reports suggesting that β blockers differ in terms of their mechanism of action and their effects on glucose and lipid metabolism with respect to their molecular pharmacological mechanisms (Ladage et al., 2013) and particularly, nonvasodilating β blockers are associated with a worsening of glycaemic and lipidic control at rest (Fonseca, 2010). With regard to α blockade, 100 mg labetalol did not produce significant differences in resting and post-exercise HR. This is in line with previous studies showing that labetalol at doses of 100, 200 and 400 mg did not alter resting HR compared to placebo in healthy males (Beachen et al., 2002). However, little evidence has indicated a dose-dependent reduction in post-exercise HR at 1 and 2 hours (Tham et al., 1993).

The present findings suggest that combined α/β-adrenergic blockade during moderate-intensity exercise does not influence appetite/satiety sensations or subsequent food intake following exercise in obese women. The changes in blood glucose and plasma FFA suggest 100 mg of α/β-adrenergic blocker was sufficient to induce β-adrenergic blockade. Labetalol was chosen as a safe and well understood α/β blocker; however, it has greater affinity for β- than α-adrenoceptors (MacCarthy and Bloomfield, 1983). For this reason, any conclusions with respect to α-adrenoceptor blockade should be drawn with caution. Labetalol decreased circulating FFA and increased glucose concentrations, which indicate inhibition of catecholamine-stimulated lipolysis and confirm the primarily β-adrenoceptor blockade. There is no simple way to know if α blockade was adequate. There is evidence that attributes the anorexigenic effect of catecholamines to α-adrenoceptors in the brain (Wellman et al., 1993). It is this effect that a popular class of anti-obesity drugs exploits to reduce eating behaviour (e.g., sibutramine) by blocking NA reuptake through activation of brain α_1_-adrenoceptor receptors (Lean, 2001).

### Study limitations

The monitoring period of appetite response was relatively brief in our study. According to recent findings, changes in appetite hormones could emerge over the following 24 hours (King et al., 2015). Determining the EI response might also require multiple ad libitum meals, rather than single feeding episodes (Deighton and Stensel, 2014). In our study, all women were premenopausal but menstrual cycle was not controlled for in the study design to account for the perceived confounding effect of the menstrual cycle on appetite sensations, appetite-regulating hormones and EI (Brennan et al., 2009). However, we did not find any differences in appetite responses and EI between the interventions, which could have been confounded by cyclical changes in sex hormones in our women.

## Conclusions

In conclusion, inhibition of exercise-induced adrenergic activity by combined α/β-adrenergic blockade and moderate increases in adrenergic activity induced by intravenous adrenaline infusion did not significantly affect acute appetite ratings or ad libitum intake in obese premenopausal women. Testing with a more potent α blockade may be necessary to trigger a detectable effect and elucidate the role of adrenergic activity in exercise-induced anorexia. In this way we could conclude with complete confidence that the observed anorexic effect of exercise on appetite in obese women is not mediated by increased adrenergic activity. Finally, to definitively exclude sympathetic system involvement in exercise-related appetite regulation, the effects of more selective α-adrenergic stimulation on leptin-mediated appetite sensitivity after exercise should be investigated.

## Supplemental material

Suppplementary_files - The effects of moderate alterations in adrenergic activity on acute appetite regulation in obese women: A randomised crossover trial Click here for additional data file. Suppplementary_files for The effects of moderate alterations in adrenergic activity on acute appetite regulation in obese women: A randomised crossover trial by Fotini Tsofliou, Yannis P Pitsiladis, Jose Lara, Marios Hadjicharalambous, Ian A Macdonald, Mike A Wallace and Mike E J Lean in Nutrition and Health
